# Adverse effects of sterilization processes on the fundamental topographic properties of modified dental implant surfaces

**DOI:** 10.1007/s10856-024-06813-y

**Published:** 2024-07-29

**Authors:** Marcel F. Kunrath, Roberto Hubler, Christer Dahlin

**Affiliations:** 1 Department of Biomaterials, Institute of Clinical Sciences, Sahlgrenska Academy at University of Gothenburg, P.O. Box 412, SE 405 30 Göteborg, Sweden; 2 School of Health and Life Sciences, Post-Graduate Program in Dentistry, Pontifical Catholic University of Rio Grande do Sul, Porto Alegre, RS Brazil; 3 School of Technology, Post-Graduate Program in Materials Technology and Engineering, Pontifical Catholic University of Rio Grande do Sul, Porto Alegre, RS Brazil

## Abstract

The employ of sterilization processes are essential to investigate biomaterials aiming for experimental, preclinical, or clinical applications with biological tissues. However, responsive surface properties of biomaterials may be susceptible to sterilization processes, compromising important physio-chemical characteristics. For that reason, this in vitro study aimed to investigate the effects of three different processes for sterilization (humid heat under pressure, UVC-light exposure, and Gamma irradiation) on the major topographical properties of implant surfaces applied to dental bone-anchored implants and/or implant-abutments. Three groups of implant surfaces were developed: a smooth machined surface, a micro-texturized surface, and a hydrophilic micro-texturized surface. The implants were sterilized with three methodologies and characterized regarding surface morphology, elemental surface composition, roughness parameters, wettability characteristics, and compared to the samples as-developed. Surface morphology and roughness parameters were not modified by any of the sterilization processes applied. On the other hand, hydrophilic implants were negatively affected by autoclaving. After package opening, hydrophilic features showed to be sensible to atmospheric air exposition independently of the sterilization process performed. Our findings revealed significant chemical changes on the implant surfaces caused by autoclaving and UVC exposure; additionally, the results showed the importance of selecting an appropriate sterilization method when investigating hydrophilic implants so as not to generate imprecise outcomes.

**Graphical Abstract:**

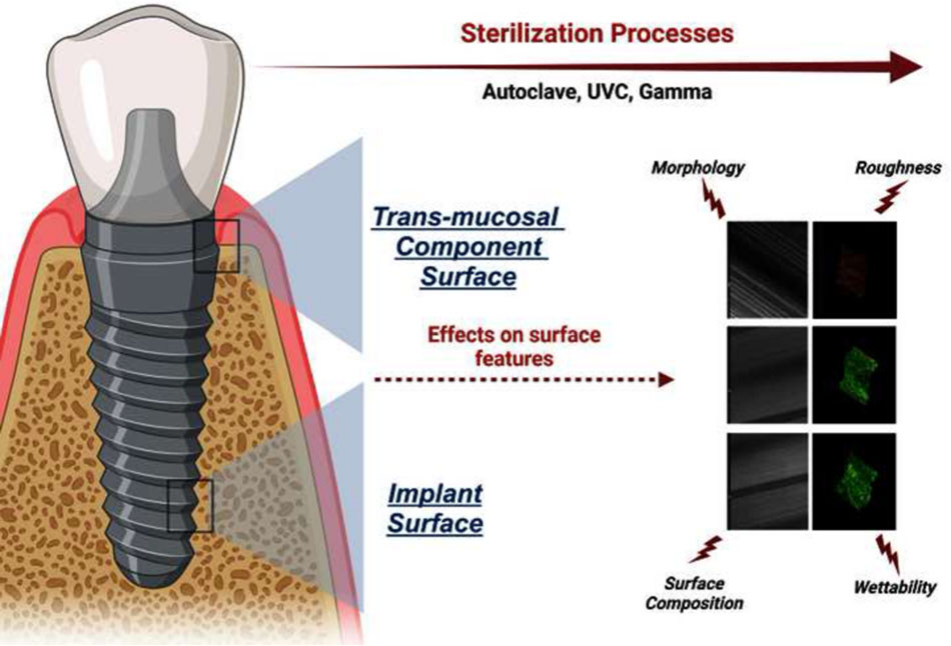

## Introduction

Dental implants have been modified according to their macro design, topography, and chemical composition to promote faster and improved osseointegration for patients needing oral treatments [1–3]. Complex surface modifications such as hydrophilization and micro-nano-texturization have been applied in preclinical and clinical studies aiming for rapid cellular interaction and accelerated bone formation around the implants [4–6]. When inserted in patients, these implant surfaces interact with blood proteins and/or bone-related cells and their as-manufactured properties cannot be negatively altered by previous procedures.

Sterilization is an essential process for biomedical materials that aim for the total removal of microorganisms, preventing possible infections/failures [7, 8]. Consequently, a sterilized biomaterial/surface exposes to the tissues/cells your biocompatible chemical composition without interference of external microorganisms, promoting optimistic conditions for bio-integration in the human body or animal models. Several processes for sterilization have been suggested and applied in biomedical implants for preclinical investigations, clinical studies, and by companies in commercially available implants [7–13]; for instance, physical, chemical, or physio-chemical methods can be cited: heat processes [10], light irradiation [11], cold plasma application [12], humid heat under pressure [13], ethylene oxide [13], and Gamma irradiation [13]. Normally, the above-mentioned processes, have presented successful results for combating bacterial contamination. However, some studies have shown that processes for sterilization may impair or change some implant surface properties [11, 14, 15]. Nanotextured surfaces were demonstrated to be damaged after autoclave sterilization (both dry and wet) in their morphological structure [14], furthermore, ultraviolet-light (UV) applications have been shown to modify wettability conditions [16], and the application of UV-irradiation for disinfection showed to promote higher protein absorption and cell proliferation compared to chemical and autoclaving process [17].

Hydrophilicity of the implant surface has been reported to accelerate cell attachment, cell spread, and early bone mineralization [18, 19]. Therefore, hydrophilic implants must arrive at the surgical site without any alteration in their surface conditions. Two situations may be associated with loss of hydrophilicity after the manufacture of implants; one is regarding the handling by dental surgeons, which might interact with contaminants, saliva, or exposure to the atmospheric air for long periods of time compromising the hydrophilicity previously to the implant insertion [5, 20, 21]. Another issue may be related to the sterilization processes applied before the clinician’s manipulation, and the storage methodology selected by the company/researcher that would impair this property; furthermore, the possibility of damages on sterilized packages storing implants after selling might affect surface properties.

Considering these susceptible features of the current modified dental implants, it is not clear in the literature which surface properties may be affected by sterilization processes and significantly alter as-manufactured properties. Therefore, this experimental study aimed to investigate the effects of three different sterilization processes on distinct modified-implant surfaces manufactured for application in dental implants and/or dental abutments. Consequently, qualitative and quantitative topographical characteristics before and after sterilization processes were evaluated.

## Materials and methods

### Implants and surface treatments

A total of 246 screw-shaped pure Titanium (Neoss Ltd, London, UK) implants, 2 mm in diameter and 2.3 mm in length were manufactured for this investigation. The implants were divided into three different groups (*n* = 82/per group) regarding the surface treatment performed.

Group 1: a smooth machined surface, as a control.

Group 2: a two-steps acid-etched surface, micro-texturized surface.

Group 3: a two-steps acid-etched surface + hydrophilic treatment, hydrophilic micro-texturized surface.

Regarding the surface treatments, in brief, group 1 (smooth machined surface) was used in the experiments as received by the manufacturer; the implants were only cleaned in an ultrasonic bath with a sequence of detergent, alcohol 70%, and deionized water (DI), followed by drying in a controlled hood. For the group 2, acid etching surface treatment was performed using 4.0% hydrofluoric acid (HF) immersion for 2 min, washing with DI for 15 min in ultrasonic bath for acid neutralization, followed by 70% diluted sulfuric acid (H_2_SO_4_) immersion for 20 minutes and 80° Celsius, additionally; the implants were cleaned in an ultrasonic bath applying the same protocol applied to the group 1. For hydrophilicity activation, a post-treatment was performed; group 3 was exposed to a 15 W bactericidal lamp delivering a mixture of irradiation via a single source (λ = 250 nm and λ = 360 nm) for 1 h following a previously published study [22]. The samples were immediately after UV-activation immersed in DI and stored in the dark to simulate hydrophilic commercial implants and defend surface properties. A triplicate (n = 3 per group/per analysis) was used for previous characterization to the sterilization processes and the results were applied as control parameters.

After surface treatments, the samples were sealed in sterilized packages and stored in the dark at same conditions (20 °C in a controlled hood) for one week until subsequent start of sterilization processes.

### Sterilization processes

For implants sterilization, three different processes were performed as described below and recommended by the manufacturers:

Process 1: Humid heat under pressure sterilization, Autoclave (Varioklav^**®**^, HP Labortechnik, Oberschleissheim, Germany) using a protocol of 60 min/134**°** Celsius under 206 kPa.

Process 2: UVC-light exposure in a clean chamber (UVC LED System, ProPhotonix, NH, USA; lamp of 265 nm wavelength - peak irradiance of 90 mW/cm^2^ and peak energy density (dose) of x 90 mJ/cm^2^) for 30 min.

Process 3: Gamma irradiation (Gamma irradiation by Bioster, Veverská Bítýška, Czech Republic—Radioisotope Co60 is applied as the radiation source and sterilization dose 25 kGy).

**Fig. 1:**
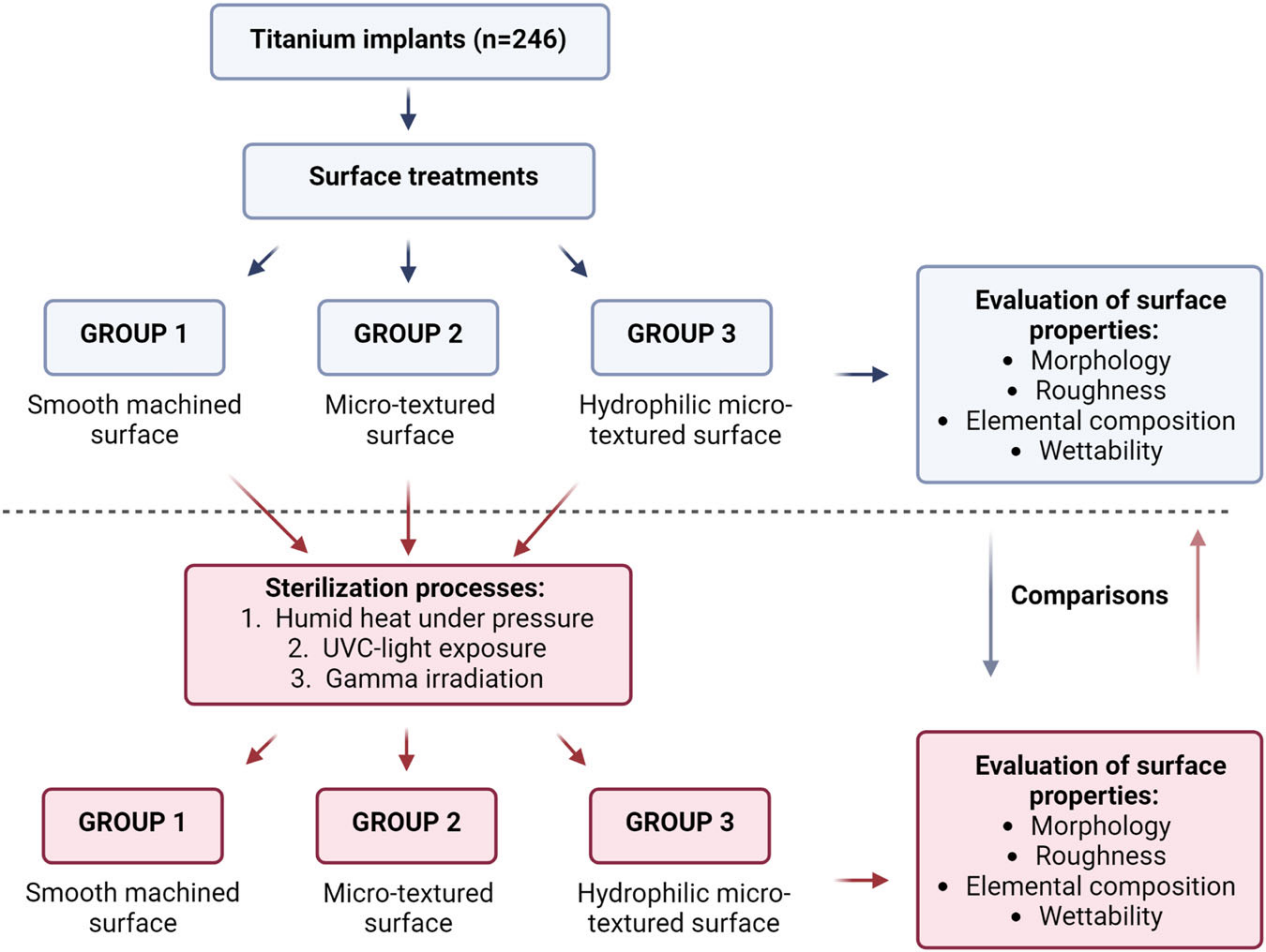
Study flowchart describing step-by-step the methodologies applied to this study. Created with Biorender

After sterilization processes, the samples were immediately submitted to the characterization assays, as described in the flowchart (Fig. 1).

### Characterization

#### Surface morphology and chemical composition

For qualitative surface morphology investigation, a scanning electron microscope (SEM, JEOL JSM-7800F Prime FEG) coupled with energy dispersive X-ray (EDX, Oxford, UK) spectroscopy was used. Three samples of each implant group were investigated before and after the sterilization processes. Implants were removed from the sterilized packages using sterilized tweezers and directly fixed on the SEM table with double sided tape to prevent any contamination. High and low magnifications (200x - 1000x) on SEM were applied after the sterilization processes to investigate tentative qualitative morphological changes on the surfaces. For elemental surface composition analysis, three different spots were analyzed using EDX in each implant per group (*n* = 3) to identify possible surface changes in quantitative chemical composition.; the evaluated percentages were described as an average with standard deviations.

#### Roughness parameters

To investigate roughness parameters on the different implant groups, (*n* = 3 per surface treatment/per sterilization process), an atomic force microscope (AFM, Dimension Icon, Bruker, Massachusetts, USA) was applied. The NanoScopeAnalysis® software was used to create 3D-graphs and investigate two different parts of the screw-type implants (valleys and tops) and three different parameters (Sa = arithmetical mean height surface, Sq = root mean square height, Sdr = developed interfacial area ratio), using a cut-off value of 30 μm [23]. Additionally, three different surface spots for verify consistence of surface treatment accordingly to previous recommendations [24].

#### Surface wettability

For wettability tests, a goniometer was used for dropping DI over the implants (droplets of 10 μl), before and after sterilization processes (*n* = 3 per surface/per sterilization process). The captured images were transferred to ImageJ software (LOCI, University of Wisconsin, USA) to measure the contact angle (degrees) between the implant surface and DI dripped, using a “contact angle measurement plug-in” associated with the specific software.

The measurements were performed prior to any sterilization processes (positive control) and immediately after the sterilization processes. Additionally, to investigate the possible variations caused by sterilized-package opening and interaction with the external environment on the wettability measurements, the screw-type implants were removed from the sterilized packages and exposed to normal air conditions (24° Celsius/ atmospheric air) in a laboratory area to investigate the short-outcomings (1, 5, 10 min) and the long-outcomings (24 h and 1 week) of exposition to atmospheric air, then, additional measurements were performed to analyze the wettability conditions.

### Statistical analysis

Data are exhibited as mean ± standard deviation (SD). Experiments were performed in triplicate. A one-way analysis of variance ANOVA followed by post hoc testing (Tukey HSD) was applied to elemental composition, roughness parameters, and wettability comparisons using the software (GraphPad Prism 9.0, California, USA). To check data distribution, Shapiro-Wilk test was applied; to check data dependence, Durbin-Watson test was used. A significant variation between groups was considered to occur at 5% (*p* < 0.05).

## Results

**Fig. 2:**
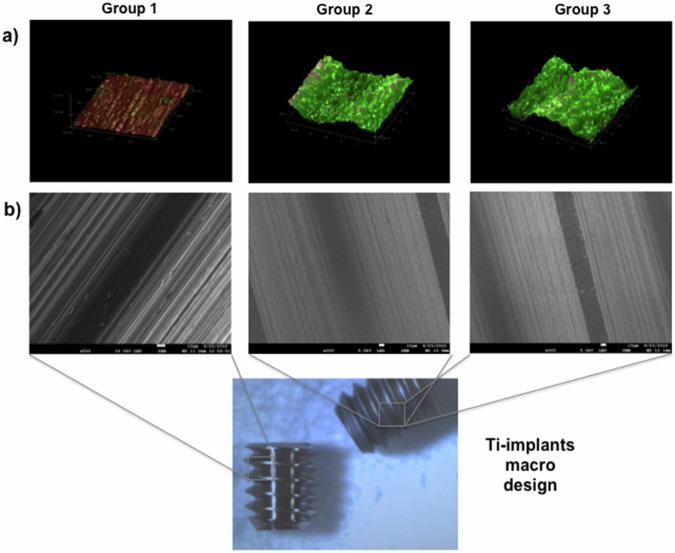
Illustrations showing qualitative images of the developed macro implants and their surfaces. Group 1: smooth machined surface; Group 2: micro-texturized surface; and Group 3: hydrophilic micro-texturized surface. **a** 3D graphs generated by atomic force microscopy (AFM) showing qualitative roughness characteristics of the three implant groups (AFM cut-off value applied: 30 μm). **b** Scanning electron microscopies (scale bars—10 μm) showing surface morphologies immediately after the different surface treatments (without sterilization processes applied), and image demonstrating the Ti-implants macro-design

As a result of the proposed surface treatments on the implants, three different surface groups were exhibited showing distinct physio-chemical characteristics. Group 1: smooth machined surface; Group 2: micro-texturized surface; and Group 3: hydrophilic micro-texturized surface. It was possible to notice topographical qualitative differences between group 1 compared to groups 2 and 3 using AFM and SEM; additionally, it was verified the similarity of group 2 and group 3 accordingly to topography due to non-visible alterations caused by the hydrophilic treatment applied to group 3 (Fig. 2).

### Surface morphology and surface chemical composition after sterilization processes

**Fig. 3:**
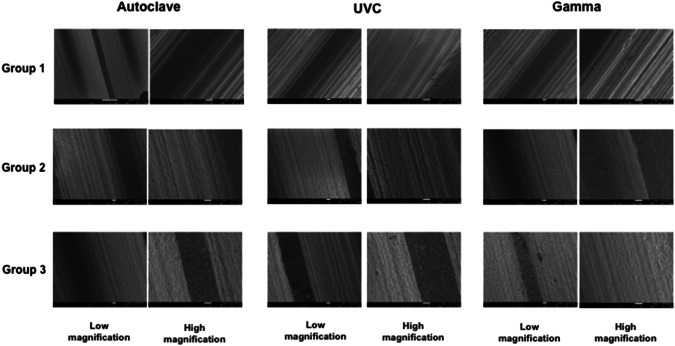
Scanning electron microscopies showing the qualitative morphological aspects of the three different implant groups without visible alterations caused by the sterilization processes. Scanning electron microscopies covered distinct spots such as valleys and threads from the different implant samples. Low magnifications were considered (200x–550x) and high magnifications (×1000)

Scanning electron microscopies using high and low magnifications were registered from different spots (threads/valleys) on the three implant groups after the proposed sterilization processes. The qualitative images showed non-significant alterations regarding morphological characteristics for any type of surface treatment or caused by sterilization processes (Fig. 3). The surface morphologies totally corroborate with the surface morphologies registered previous to the sterilization processes as can be verified comparing SEM images presented in Figs. 2 and 3 for the three surface groups.

On the other hand, EDX investigation revealed important changes in the elemental percentages after sterilization processes compared to control implants. Autoclaved implants showed slightly higher values for Carbon elements for all groups analyzed, suggesting an effect of humidity and heat treatment on chemical composition present on the surface. Additionally, the UVC process reduced the levels of Carbon for all the groups analyzed compared to their controls, suggesting a removal effect on the surface appearance of Carbon atoms. Group 3 revealed susceptibility for elemental changes after sterilization processes (Table 1).

**Table 1:** Atomic ratio (at/ %) per surface group previous to sterilization processes (control) and after different sterilization methodologies

Groups/Chemical elements	Titanium	Oxygen	Carbon
Group 1 (control)	62 ± 5%	28 ± 2.2%	10 ± 2%
Group 1 after autoclave	59 ± 5.5%	26 ± 3%	15 ± 2%
Group 1 after UVC	60 ± 7%	38 ± 4.2%	2 ± 0.4%^*^
Group 1 after Gamma	70 ± 5.5%	22 ± 2.4%	8 ± 1.2%
Group 2 (control)	75.5 ± 8.4%	14.5 ± 1.2%	10 ± 2%
Group 2 after autoclave	73.5 ± 7%	13.5 ± 2%	13 ± 2.4%
Group 2 after UVC	80 ± 10.2%	20 ± 5%	0%^*^
Group 2 after Gamma	68.5 ± 8%	22 ± 4%	9.5 ± 4%
Group 3 (control)	30.5 ± 5.4%	64.5 ± 12%	5 ± 1%
Group 3 after autoclave	52.5 ± 10%^*^	30 ± 8%^*^	17.5 ± 5%^*^
Group 3 after UVC	50.5 ± 8%^*^	49.5 ± 10.2%	0%^*^
Group 3 after Gamma	35 ± 6%	49 ± 8%	16 ± 3%^*^

Obs: ^*^ symbolizes altered percentages statistically significant (*p* < 0.05) compared to the control parameter of the same group

### Roughness parameters

**Fig. 4:**
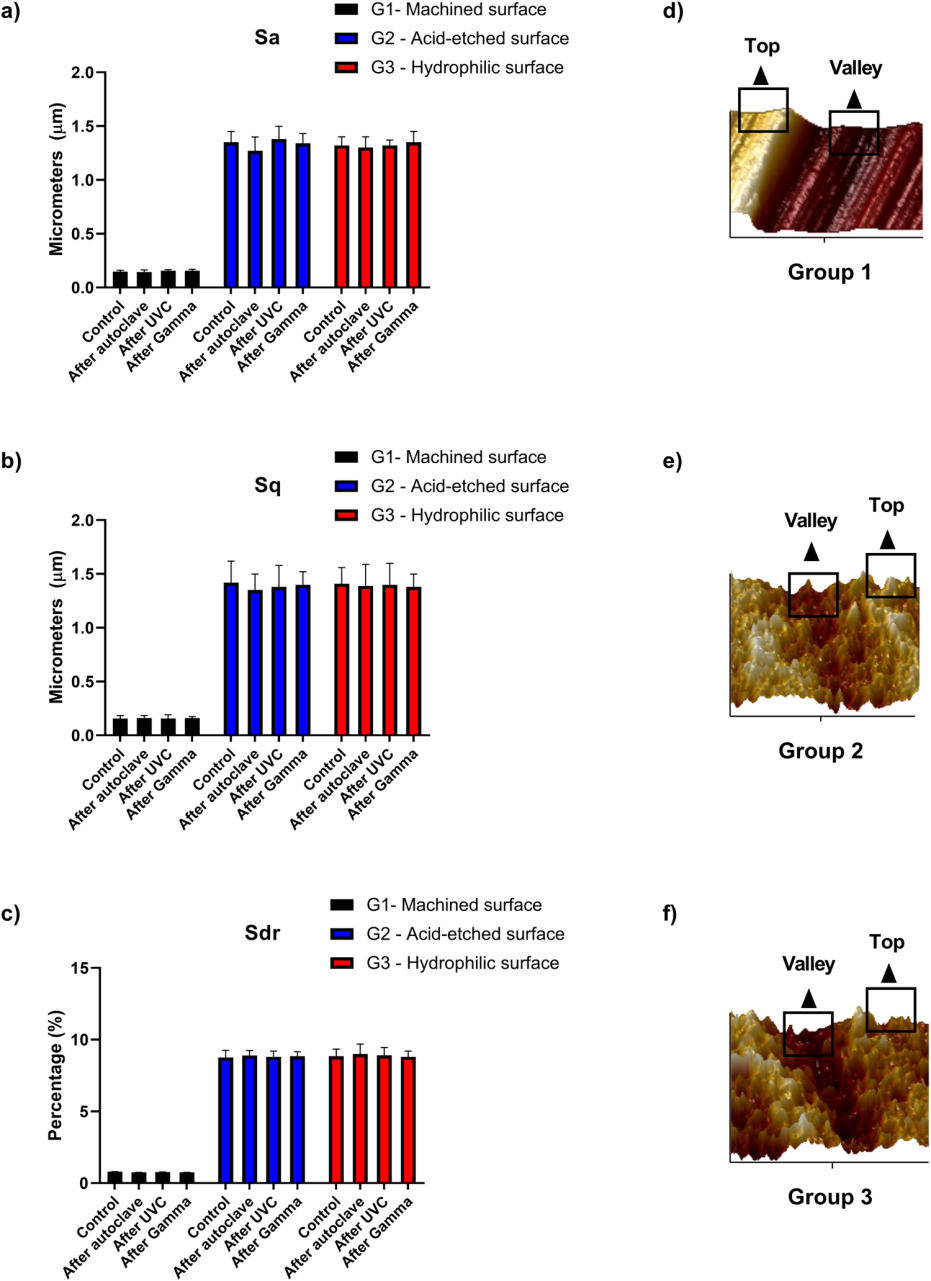
Graphs revealing the roughness parameters investigated prior to sterilization processes (control) and after the sterilization processes. **a** Sa = arithmetical mean height surface; (**b**) Sq = root mean square height; (**c**) Sdr = developed interfacial area ratio. Stable parameters were noticed without alterations caused by sterilization processes. **d**–**f** Representative illustrations of implant areas (tops and valleys) investigated to acquire the roughness data

Before and after sterilization processes, roughness characteristics showed to maintain stable values for bi-dimensional and three-dimensional roughness parameters without significant alterations (Fig. 4).

### Wettability

**Fig. 5:**
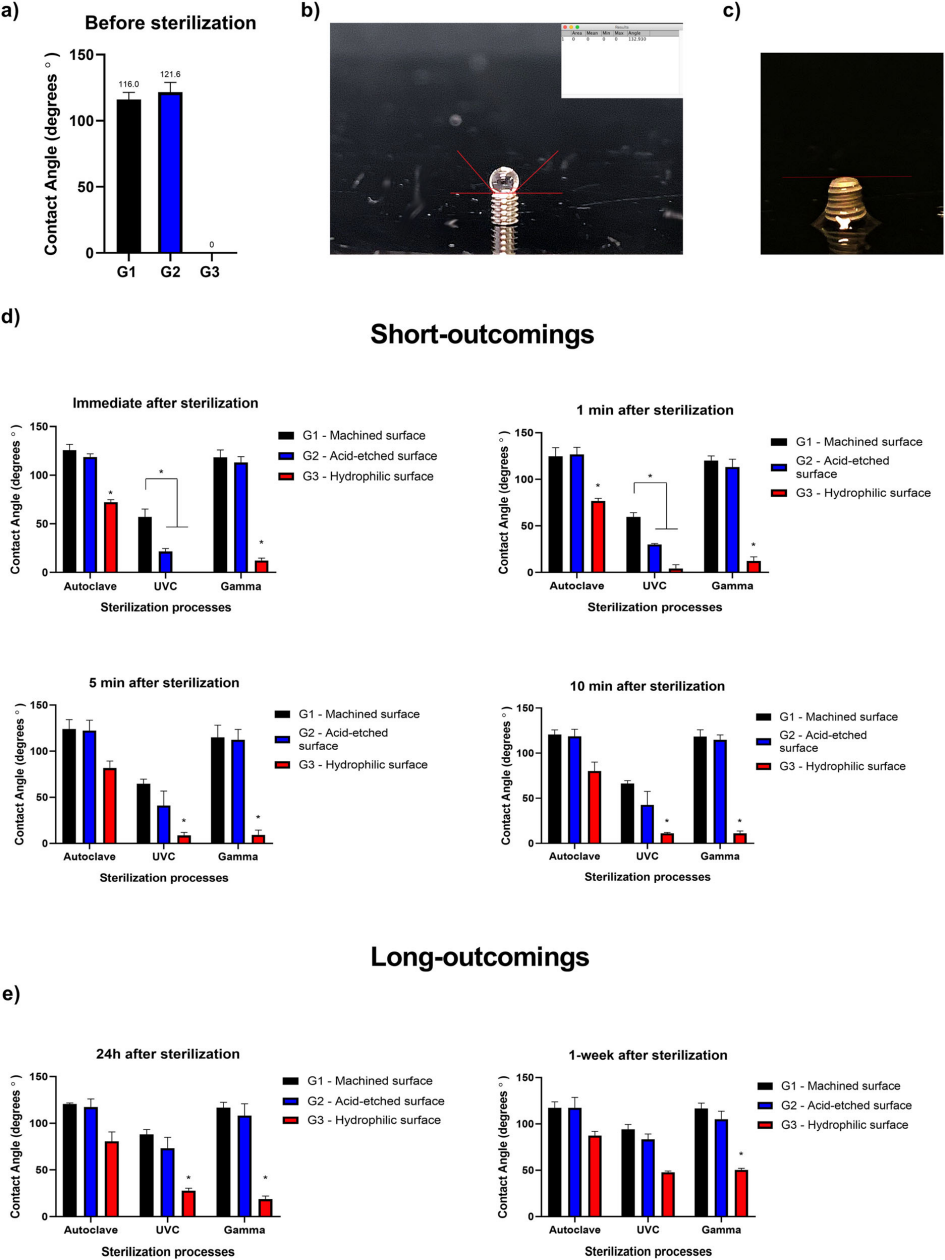
Wettability investigation. **a** Contact angles measured immediately after surface treatments. **b** Example of methodology applied to investigate the wettability on the implant surfaces showing a hydrophobic status. **c** Example of methodology applied to investigate the wettability on the implant surfaces showing a hydrophilic status – 0° contact angle. **d** Graphs representing the wettability measurements immediately after opening the implant sterilized-packages and after 1, 5, and 10 min of exposition to atmospheric air (short-outcomings). **e** Wettability measurements after 24 h and 1-week of package opening and exposition to atmospheric air (long-outcomings). * Symbolizes statistical significance (*p* < 0.05) difference from other groups

Immediately after surface treatments, group 3 demonstrated highly hydrophilic properties with a contact angle measured 0.0 degrees, while groups 1 and 2 showed hydrophobic conditions (Fig. 5a–c). Immediately after the three different processes for sterilization was noticed notable changes in wettability features for group 3 after applying humid heat under pressure sterilization and for groups 2 and 3 after applying UVC-sterilization. Humid heat under pressure sterilization compromised significantly (*p* < 0.05) the hydrophilicity in group 3 (contact angle changes: 0.0 degrees to ~60 degrees) compared to the control measurements prior to the sterilization processes. Furthermore, the UVC-sterilization maintained the hydrophilicity in group 3 and activated hydrophilic conditions in group 2 (contact angle changes: ~121 degrees to ~21.1 degrees).

The short-outcomings (1–10 min) of exposition to the atmospheric air did not show statistically significant alterations between the time points investigated, however, it was possible to notice a slight increase in contact angle values for groups 2 and 3 after UVC sterilization (Fig. 5d).

After 24 h of exposition to atmospheric air, groups 2 and 3 showed higher values for contact angles in the samples sterilized with UVC-light and Gamma irradiation, however, still demonstrated hydrophilic characteristics with contact angles around ~28.5 degrees for UVC-light and ~21.2 degrees for Gamma irradiation. After 1-week of atmospheric air exposition, implant surfaces demonstrated to lose hydrophilic conditions modifying to intermediate wettability (Fig. 5e). Significant statistical reduction (*p* < 0.05) of hydrophilicity was observed comparing short-outcomings (0–10 min) to long-outcomings (24 h and 1 week) for groups 2 and 3 after UVC process; and for group 3 using Gamma irradiation after 1 week.

## Discussion

Several methodologies have been proposed to modify dental implant surfaces in the current implant market [2, 3, 25], moreover, innovative surface treatments for dental implants have been applied in experimental and preclinical research models aiming to demonstrate advances in the osseointegration process and for preventing peri-implant diseases [2, 3, 5, 25–29]. In this study, we developed three different implant surfaces showing different surface topographies: a smooth machined topography (commonly applied on implant abutment surfaces), a micro-textured topography, and a hydrophilic micro-textured topography, simulating the prevailing surface characteristics exhibited on the implant commercial market. Among the several surface properties modified by researchers and industry, hydrophilicity has been reported as an important property to stimulate protein absorption and promote faster cell spread/differentiation on dental implant surfaces [30, 31], on the other hand, studies have demonstrated the sensibility of hydrophilic surfaces to atmospheric oxygen and other oral fluids [5, 18, 32]. Reinforcing these statements of a reactive surface property, our findings revealed significant susceptibility and impairment of hydrophilic properties after the autoclaving process. Applying a sterilization process using humid conditions associated with high temperature and pressure, induces changes in the surface chemistry and stimulates oxidative processes, thus, modifying wettability features.

Experimental, preclinical, and clinical studies developing or testing hydrophilic surfaces should apply a correct process for sterilization to not cause hydrophilic impairment and consequently, generate potential biased outcomes. Other studies have corroborated our results that autoclaving is influential to hydrophilic surfaces and to non-hydrophilic surfaces. For instance, Park et al. showed an increase in the contact angles (wettability) after autoclaving their experimental samples, the authors explained their results by the altered temperature and pressure applied in the autoclaving process, affecting the oxidation states on the surface [7]; Junkar et al. revealed that autoclaving may change structural characteristics of implant surfaces covered by TiO_2_ nanotubes, suggesting susceptibility of the nanostructured surfaces to autoclaving processes due to alterations in morphological topography [33]; and Shi et al. demonstrated reduced crystallinity and hydrophilicity loss after conventional autoclaving induced by altered temperature and pressure [34].

UV-light treatments have been reported to improve and activate hydrophilic characteristics by several authors [18, 32, 35, 36]. Also, the application of methodologies using UV-light processes has shown significant antibacterial effects on different types of surface topographies and demonstrated to inhibit several bacteria species [36]. However, studies have demonstrated multiple instruments to perform the application of UV irradiation and various protocols such as: variability on the time application; distinct wavelength; different devices as radiation sources [18, 32, 35–37], moreover, some authors defend the hypothesis that UV-light application may be only considered a disinfection surface process and its successful result is time-dependent [38, 39]. In the present study, the application of UVC treatment demonstrated a significant impact on the wettability properties and on the surface chemistry. Significant changes were noticed on the surface chemistry such as: Carbon ratio (%) reduction and Oxygen ratio (%) increase, hereby promoting a reactive surface and improving the hydrophilic features. Nevertheless, the absolute protocol for the application of UV-light treatments for dental implant surfaces aiming to combat the large spectrum of bacteria including the anaerobic species still is not totally clear in the literature. Gamma irradiation is reported as an appropriate sterilization method for biomaterials due to not increasing temperatures during the entire process [7, 39]. Our experiments corroborated the previous positive statements about Gamma irradiation sterilization, not revealing any significant alteration in morphological characteristics, roughness parameters, or wettability conditions.

Interestingly, a previous study demonstrated slight changes in the roughness parameters of micro-texturized implant surfaces after sterilization with autoclave and UV-light application [7]. Our investigation did not find any significant alteration on bi-dimensional or three-dimensional parameters of roughness, suggesting that topographical alterations caused by sterilization processes might be surface-topographic-dependent influenced by micro- nano-scaled morphologies or by the base-materials applied to manufacture the implants.

Corroborating previous studies investigating hydrophilicity maintenance [18, 32, 40], our results corresponded to the loss of hydrophilic properties after opening the sterilized-packages and exposure of surfaces to environmental atmospheric air conditions (24 h and 1 week), revealing prejudice of this relevant property. Clinically, this specific outcome might suggest an important issue after opening sterilized-packages of dental implants or due to damaged packages. The surface exposure to atmospheric air may compromise the hydrophilic properties as-manufactured. Furthermore, the positive effects of superhydrophilic surfaces still are quite controversial in clinical osseointegration. While some authors have reported a significant influence of hydrophilic properties on the early clinical response of implant osseointegration [4, 41], other hypotheses have pointed out a sensible status of hydrophilic implants, suggesting that in the first surface-tissue contact the superhydrophilic conditions might immediately change and adapt to the energy status of the oral fluids in interaction, modifying the external reactive layer of the implant surface and losing the hydrophilic property [5, 42, 43].

Some limitations of this experimental study do not allow direct translation of results to a complete clinical scenario and should be taken in consideration: the implant sizes are smaller than commercially available implants; the proposed surface treatments can differ from commercial surface treatments; and the use of triplicates for the outcomes investigation generated minimal statistical power. Additionally, there are several methods to activate hydrophilicity on dental implant surfaces such as plasma application [18], chemical approaches [41], dry technologies [44], and coatings [45], which need to be tested individually in experiments and may show distinct outcomes. Further studies using commercial implants, different modified-hydrophilic surfaces, and a higher number of samples to achieve stronger statistical power are necessary to affirm the complete clinical relevance of these findings. Nevertheless, the implants developed in this investigation are similar to commercially available dental implants according to thread structuration and format, motivating suppositions significantly relevant compared to studies using flat disks. Furthermore, the scientific relevance of our findings for preclinical research using hydrophilic implants is of paramount importance to promote reliable results in future studies.

## Conclusions

Macro- and micro-morphological characteristics and roughness parameters of the implant surfaces developed were not altered by the sterilization processes. Hydrophilic properties were significantly modified by autoclaving (negatively) and UVC application (positively); Gamma irradiation showed to not compromise any surface property. Additionally, hydrophilicity maintenance may be compromised by interaction with atmospheric air after packages-opening. Taken together these results, experimental and preclinical studies investigating hydrophilic implant surfaces must select an appropriate sterilization process and adequate implant storage aiming to not compromise hydrophilic properties and subsequent research outcomes.
